# Biportal endoscopic transforaminal lumbar interbody fusion using a large cage for degenerative spondylolisthesis with stenosis

**DOI:** 10.3171/2024.1.FOCVID23231

**Published:** 2024-04-01

**Authors:** Dong Hwa Heo

**Affiliations:** Department of Neurosurgery, Endoscopic Spine Surgery Center, Chungdam Harrison Spinartus Hospital, Seoul, South Korea

**Keywords:** lumbar, spondylolisthesis, fusion, biportal, endoscopy

## Abstract

Recently, biportal endoscopic transforaminal lumbar interbody fusion (TLIF) has been used to treat lumbar degenerative diseases, such as lumbar instability and spondylolisthesis. Biportal endoscopic TLIF may have the advantages of endoscopic spine approaches and minimally invasive lumbar fusion surgeries. In this biportal endoscopic TLIF, large cages similar to oblique lumbar interbody fusion (OLIF) cages have been used. Biportal endoscopic TLIF using a large cage can be successfully performed in the lower lumbar area. The author presents the surgical technique of biportal endoscopic TLIF using a large cage.

The video can be found here: https://stream.cadmore.media/r10.3171/2024.1.FOCVID23231

## Transcript

### 0:27

I am presenting the case of a 65-year-old male patient who is experiencing severe radiating pain in both legs and buttocks, accompanied by intermittent claudication of a neurological nature. This individual faces considerable difficulty walking even a distance of 100 m.

### 0:49

Preoperative dynamic X-rays, including flexion and extension views, revealed the presence of instability and degenerative spondylolisthesis at the L4–5 level.

### 1:02

Preoperative magnetic resonance imaging (MRI): The preoperative T2 axial and sagittal images displayed evidence of degenerative spondylolisthesis and severe lateral recess stenosis at the L4–5 region.

### 2:14

I have chosen the biportal endoscopic TLIF that utilizes a larger-sized cage as the surgical intervention for this patient. This approach involves creating two portals: the first serves as an endoscopic viewing portal, while the second functions as a working portal. The procedural steps of this surgery largely align with those of minimally invasive TLIF.

### 1:40

The patient is positioned in a prone position. Jackson surgical table or Wilson frame was used as in conventional posterior fusion surgeries. Specifically, for single-level biportal endoscopic TLIF procedures, it is preferred to administer epidural or spinal anesthesia.

### 2:00

A large cage akin to an oblique lumbar interbody fusion (OLIF) cage was employed during this procedure. This larger cage’s dimensions were slightly less than those of OLIF cages yet notably larger than standard TLIF cages. I am able to incorporate this larger cage during the biportal endoscopic TLIF.

### 2:24

Under C-arm fluoroscopic guidance, two portals are created at the left-sided L4 and L5 levels. A working portal is established along the lateral border of the L5 pedicle, while an endoscopic viewing portal is established medially at the disc level of L4–5. Initially, a caudal working portal is established, followed by the insertion of serial dilators. Subsequently, a working sheath is placed at the working portal. Additionally, a trocar is inserted through a small cranial skin incision to create the endoscopic viewing portal. It’s important to note that the endoscopic viewing portal is solely used for spinal endoscopy, while the working portal is exclusively used for surgical instruments.^1,2^

### 3:19

These images serve as an overview of the biportal endoscopic lumbar approach. A 4-mm-diameter, 0° endoscope is employed in this specific endoscopic fusion technique. The surgical procedures are executed through two portals while maintaining continuous saline irrigation throughout the procedure.^1,2^

### 3:48

Laminotomy at L45 left: Initially, I conduct dissection to expose the lower lamina at the L4 level. Subsequently, a laminotomy is performed using a diamond drill and a Kerrison rongeur.

### 4:03

After laminotomy, left-side inferior articular process is cut and removed.

### 4:18

L5 upper laminar is partially removed using a diamond drill and a Kerrison rongeur for full exposure of ligamentum flavum.

### 4:37

Ipsilateral ligamentum flavum is totally removed, and ipsilateral traversing nerve root is completely decompressed. There is a noticeable presence of severe adhesion between the dura and the ligamentum flavum, visibly observed during the procedure.

### 5:05

To address the contralateral decompression of the right L5 nerve root, complete removal of the hypertrophied ligamentum flavum on the opposite side is performed. This action ensures comprehensive decompression of the central canal at the L4–5 level and bilateral L5 nerve roots.

### 5:39

Under magnified endoscopic view, I conduct a complete discectomy of the L4–5 disc and endplate preparation. Endplate preparation is carried out exclusively through the working portal using tools such as a shaver, pituitary forceps, and various types of curettes. The objective during this process is to selectively remove the cartilaginous endplate while ensuring the preservation of the osseous endplate. Notably, the capability for endoscopic endplate preparation stands as one of the advantages offered by the biportal endoscopic TLIF approach.

### 6:25

I remove superior articular process to make enough space for insertion of a large-sized cage.

### 6:29

To facilitate the safe insertion of a large-sized cage, the dura is carefully retracted medially using a dura retractor. Subsequently, a cage guidance instrument is introduced into the interbody space of L4–5.

### 6:48

I implanted a large cage measuring 15 mm in width, 11 mm in height, and 40 mm in length. The insertion of the cage was conducted with utmost care to avoid excessive retraction of the dura. The obliquely inserted cage was transversely rotated using a cage impactor for prevention of decreasing lordosis.

### 7:09

Complete decompression of the central canal and lateral recess was achieved. Subsequently, an epidural drainage catheter was inserted.

### 7:18

Postoperative MRI revealed complete reduction of spondylolisthesis and effective decompression of L4–5 stenosis.

### 7:32

The postoperative X-ray also revealed complete reduction of the spondylolisthesis at the L4–5 level, as well as successful insertion of a large-sized cage.

### 7:52

The biportal endoscopic TLIF can be accomplished through small-sized stab wounds, as in minimally invasive TLIF procedures.

### 7:51

Following the procedure, the patient experienced significant improvement in both radicular pain and claudication. The operation lasted 135 minutes with an estimated blood loss of 150 ml. The biportal endoscopic TLIF enables direct decompression of the central canal and lateral recess. Moreover, endplate preparation is accomplished without causing injury to the osseous endplate under the meticulous view provided by the endoscope. One of the notable advantages of this procedure is the faster postoperative recovery.^3,4^

### 8:33

The utilization of interbody fusion surgery as a treatment for degenerative spondylolisthesis remains controversial. Existing literature suggests that lumbar interbody fusion surgery tends to yield improved postoperative clinical outcomes, particularly in instances of spondylolisthesis accompanied by hypermobile instability and foraminal stenosis, when compared to procedures involving decompression without fusion.^5–8^

## Disclosures

The author reports no conflict of interest concerning the materials or methods used in this study or the findings specified in this publication.

## Supplemental Information

### Patient Informed Consent

The necessary patient informed consent was obtained in this study.
